# Herbicides in Water Sources: Communicating Potential Risks to the Population of Mangaung Metropolitan Municipality, South Africa

**DOI:** 10.3390/toxics11060538

**Published:** 2023-06-16

**Authors:** Innocent Mugudamani, Saheed A. Oke, Thandi Patricia Gumede, Samson Senbore

**Affiliations:** 1 Department of Life Sciences, Central University of Technology, Free State, Bloemfontein 9301, South Africa; tgumede@cut.ac.za; 2 Department of Civil Engineering, Centre for Sustainable Smart Cities, Central University of Technology, Free State, Bloemfontein 9301, South Africa or okesaheed@gmail.com (S.A.O.); senboresamson@gmail.com (S.S.)

**Keywords:** pesticide and herbicide, potential risks, water sources, ecological and health risks, Mangaung, residue

## Abstract

Pesticides are an important tool for maintaining and improving the global population’s standard of living. However, their presence in water resources is concerning due to their potential consequences. Twelve water samples from rivers, dams/reservoirs, and treated drinking water were collected from Mangaung Metropolitan Municipality in South Africa. The collected samples were analysed using high-performance liquid chromatography linked to a QTRAP hybrid triple quadrupole ion trap mass spectrometer. The ecological and human health risks were assessed by risk quotient and human health risk assessment methods, respectively. Herbicides, such as atrazine, metolachlor, simazine and terbuthylazine, were analysed in water sources. The average concentrations of simazine in rivers (1.82 mg/L), dams/reservoirs (0.12 mg/L), and treated drinking water (0.03 mg/L) were remarkable among all four herbicides detected. Simazine, atrazine, and terbuthylazine posed high ecological risks for both acute and chronic toxicity in all water sources. Moreover, simazine is the only contaminant in the river water that poses a medium carcinogenic risk to adult. It can be concluded that the level of herbicide detected in water sources may affect aquatic life and human beings negatively. This study may aid in the development of pesticide pollution management and risk reduction strategies within the municipality.

## 1. Introduction

Water resources play an important role in our daily lives as a source of water [1]. They are, however, increasingly exposed to an extensive kind of organic chemical of an anthropogenic source, such as pesticides [2]. Pesticides are chemicals or mixtures of chemicals that are primarily utilised for the protection of agricultural yields from weeds, insects, and pests, and humans from diseases [3,4]. Pesticides’ beneficial effects make them an important tool for maintaining and improving the global populations’ living standards. Each year, approximately two million tons of pesticides are utilised worldwide to control weeds, insects, and pests. Herbicides, insecticides, rodenticides, fungicides, and other pesticides are traditionally classified based on target species. Herbicides and insecticides are the primary intoxicants among pesticides. Herbicides, on the other hand, are the most commonly used type of pesticides, accounting for 47.5% of total global pesticide consumption [4]. A herbicide, according to Wang et al. [5], is any chemical, alone or in combination, whose purpose is to regulate, terminate, prevent, or alleviate the development of weeds in a crop. It is also used in forestry, community areas, parks, golf courses, and sports fields to control weeds [6].

Pesticides may contaminate water sources as a result of runoff, wastewater discharges, and return flow from agricultural and irrigated areas. They enter water through direct application to control aquatic weeds or indirectly through transportation from treated areas. Leakage and runoff from agricultural areas are the primary sources of conveyance to water sources [7,8]. The detection and quantification of pesticide in water sources together with their potential environmental health risks have been extensively documented in many areas of the world, such as in Argentina [9], Japan [10], the United States [11], India [12], the Czech Republic [13], China [14], and Malaysia [15]. In spite of such significant research outputs, the potential human health risks associated with pesticides found in various water sources essentially remain unrevealed in most of these studies. Moreover, most of these studies focus less on water sources, such as treated drinking water, which is considered the safest drinking water source. As a result of the environmental dynamics and incessant use of a number of pesticides, their presence in the water environment as well as their end products are concerning, as they may trigger possible effects on aquatic ecosystems and human health, particularly via the ingestion of water [1,10]. Pesticide exposure through water ingestion, in particular, can cause gastrointestinal and neurological effects, mimic the human body’s hormones, which reduce body immunity, disrupt hormone balance, trigger reproductive issues, pose carcinogenic effects, and reduce intelligence, particularly in children at the body development stage [4]. To protect aquatic ecosystems, regulations for the viable utilisation of pesticides have been implemented in both Europe [16,17] and America [18]. Regrettably, appropriate legislation and inspections are frequently missing in developing countries, such as South Africa, resulting in pesticide mismanagement and misuse, together with the continued use of disqualified chemicals [19].

In South Africa, approximately 26,000 tons of pesticides are used each year, with approximately 700 active chemicals registered for agricultural usage. In the African continent, South Africa adds roughly one third of all pesticides used, rendering it a high-risk country for pesticide contamination [20,21,22,23]. Monitoring pesticide levels in water resources is thus critical not only for assessing water quality but also for protecting the health of the ecosystem and South African water consumers. Previous studies on the occurrence of herbicides in South African aquatic environment found these substances in rivers in the Western Cape Province [24], dams in the Free State Province [25], seawater in the Western Cape Province [26], and wastewater influent and effluent in the KwaZulu Natal Province [27]. Hence, they did not shed light on the ecological risks of pesticides. In addition to the paucity of data on risk assessment of pesticides in various water sources, there is sparse information on the human health risks of pesticides in the country, particularly in the Free State Province (home to Mangaung Metropolitan Municipality) where almost fourteen percent (14%) of profitable agri-business in the country occurs [28]. The intensive agricultural activities in this area encourage the use of agricultural chemicals. Trizine herbicides, such as atrazine, simazine, and terbuthylazine, and chloroacetanilides herbicides, such as metolachlor, were targeted in this study because agriculture dominates the landscape of the Mangaung Metropolitan Municipality. These compounds are also the most commonly used around the world and are associated with serious health effects [4].

The widespread utilisation of agricultural chemicals, such as herbicides, in the Mangaung Metropolitan Municipality prompted this study. Here, we report the first assessment of herbicides in water sources around the Mangaung Metropolitan Municipality in the Free State Province of South Africa. To ensure good surface and drinking water quality within the Mangaung Metropolitan Municipality, it was critical to assess potential pesticide adverse effects on aquatic ecosystems and human health. To date, there is a scarcity of studies in the Mangaung Metropolitan Municipality that provide a comprehensive assessment of pesticides and their potential environmental health risks across a variety of water sources (rivers, dams/reservoirs, and treated drinking water) that are used by thousands of people for various purposes. Thus, in this metropolitan, more detailed risk assessment studies of pesticides in rivers, dams/reservoirs, and treated drinking water are desperately needed for water and human health security. Therefore, this project will fill in such a gap by determining the concentrations of herbicides in such water sources. The secondary goal is to assess the ecological, non-carcinogenic, and carcinogenic risks associated with pesticides exposure. The novelty of this study stems from the fact that it is the first to report on the environmental and human health risks of pesticides in rivers, dams/reservoirs, and treated drinking water within the Mangaung Metropolitan Municipality. The findings of the study may help to notify the community and relevant stakeholders on the environmental and public health risks of pesticide exposure. Furthermore, it will guide potential interventions to curtail pesticide pollution and concomitant risks in the region and throughout the country.

## 2. Materials and Methods

### 2.1. Study Area

**Figure 1:**
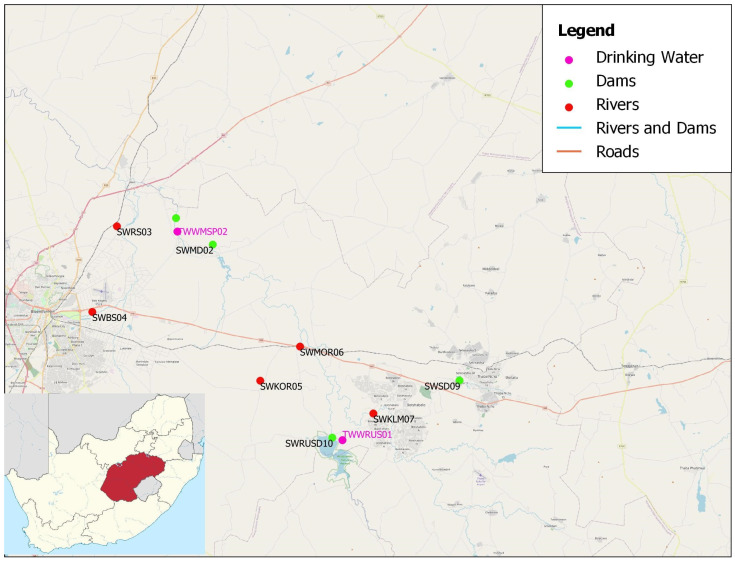
Mangaung Metropolitan Municipality.

The Mangaung Metropolitan Municipality is classified as a category-A municipality. It is centrally positioned in South Africa’s Free State Province (Figure 1). It is located at 29°10′00″ S and 26°1′67″ E, at an area that covers approximately 9899 km^2^. It is home to 878,834 people, which accounts for roughly 28% of the provincial population. The temperatures may vary from 1.3 °C to 21 °C during winter and from 13 °C to 30.9 °C during summer. The municipality’s average annual rainfall is approximately 476 mm, with February being the wettest month. The Mangaung Metropolitan Municipality has only one biome, grassland, which covers the entire municipality. In the Mangaung Metro, commercial agriculture accounts for 22.96% of land, with pivot irrigation accounting for 1.4% and cultivated orchards accounting for 0.02%. Agriculture is a major economic activity in the Mangaung Metro, with commercial, small-scale, and subsistence farming all practiced. An estimated 46,172 households, or 19.4% of the total households in the municipality, rely on agricultural activities for a living. Crop farming accounts for 67.5% of agricultural activities in the Mangaung Metro, followed by livestock farming, mixed farming, and finally other types of agriculture. Community services, finance, trade, transportation, and manufacturing are some of the other economic sectors in the municipality. As both the Water Services Authority and the Water Service Provider, the Mangaung Metropolitan Municipality is obligated to fulfil its mandate of providing safe and dependable portable water to its consumers. The Mangaung Metro’s water resources include dams (reservoirs), rivers, wetlands, and groundwater resources. The city’s bulk water supply is currently 31% supplied by its water treatment works and 69% supplied by other providers [29].

### 2.2. Sample Collection and Analysis

In September 2022, the sampling campaign was launched. Rivers, dams/reservoirs, and water treatment works (WWTW) in the Mangaung Metropolitan Municipality were targeted during the campaign. As shown in Table 1, twelve (12) water samples were collected from rivers (5), dams/reservoirs (5), and treated drinking water (2). When choosing a sampling location, accessibility, site representation, and pollution sources were all factors to consider. To collect grab water samples, 750 millilitre (mL) hygienic glass bottles with screw caps were used. During the sampling campaign, cooler boxes, ice cubes, and tags were also purchased to protect and label the samples. The 750 millilitre (mL) glass bottles were washed several times with clean water or river water prior to sampling. Furthermore, before taking the treated drinking-water sample, the tap was opened and permitted to run freely for a few minutes. Following fieldwork, all collected samples placed in a cooler box packed with ice cubes were conveyed to the laboratory, where they were stored at 4 °C until analysis.

In the laboratory (Bloemfontein, South Africa), the samples were filtered through glass fibre filters to remove particulate matter before being concentrated at a flow rate of 5 mL/minutes (min) onto methanol conditioned C18-6 mL solid phase extraction cartridges (Strata, Phenomenex, Torrance, CA, USA). The bound sample was slowly eluted from the dried cartridges with 2 mL methanol and 2 mL ethyl acetate. The eluant was vacuum dried until almost dry (Thermo Scientific Savant Speedvac, Waltham, MA, USA) and reconstituted in 1 mL purified water.

High-performance liquid chromatography linked to a QTRAP hybrid triple quadrupole ion trap mass spectrometer was used to analyse the water samples. Analyst 1.5 (AB SCIEX) software was used for all data acquisition and processing. Positive and negative ionisation modes were used to analyse the samples. During analysis, 20 microliter (μL) of each extracted sample was separated on a C18 (150 mm × 4.6 mm, Gemini NX, Phenomenex) column at a flow rate of 300 μL/min using a 5 min gradient from 5% solvent A (H_2_O/0.1% formic acid) to 95% solvent B (MeOH/0.1% formic acid) with a total run time of 9 min and 10 min in positive and negative ionisation modes, respectively, to allow for column re-equilibration. Eluting analytes were electrospray ionised in the TurboV ion source with a heater temperature of 500 “°C” to evaporate the excess solvent, 40 psi nebuliser gas, 40 psi heater gas, and a curtain gas of 15 psi. In positive ionisation mode, the ion spray voltage was set at 5500 V, while in the negative ionisation mode, it was set at −4500 V.

Pesticide analyses were carried out using multiple reaction monitoring transitions per analyte. The quantifier was the peak area on the chromatogram generated by the first and most sensitive transition, while the qualifier was the peak area generated by the second transition. The qualifier served as an additional level of confirmation for the analytes’ presence. The retention times for these two transitions must be the same as those listed in Table 2.

To validate instrument performance, the selectivity, linearity and limit of quantification (LOQ) were taken into account. Samples were submitted in batches with solvent blank runs between each sample analysed and quality control samples of known concentration interspersed. For each analyte, a four-point calibration curve with a linear fit through the origin was generated, ranging in concentration from 0.001 ppm to 1 ppm. The linear fit yielded a correlation coefficient (r) value above 0.98. Furthermore, the quantification limits ranged from 0.0001 mg/L to 0.01 mg/L as shown in Table 3.

### 2.3. Environmental Risk Assessment

The potential ecological risks of herbicides in water sources were evaluated by the environmental risk quotient (*RQ*) method. The risks were evaluated based on acute and chronic toxicities, which measure the toxic effects on the most vulnerable organisms, such as algae, invertebrate, and fish [30]. Using Equation (1), the risk quotient values were computed by comparing the measured environmental concentration (*MEC*) and the predicted no effect concentration (*PNEC*):

(1) $$RQ=\frac{MEC}{PNEC}\;$$

When performing a risk analysis for a specific contaminant and aquatic organism, i.e., algae, invertebrate and fish, it was crucial to estimate the level of contaminant in the water sources as well as the toxicity of a particular contaminant to an organism in the water sources (Table 4). The predicted no effect concentration was calculated by dividing the acute or chronic toxicity value by an assessment factor (*AF*). The toxicity results were corrected by an assessment factor during the process of determining the predicted no effect concentration of a specific herbicide in water sources as shown in Equation (2). Acute toxicity was considered the median lethal concentration (*LC*_50_) or mean effective concentration (*EC*_50_), where *AF* = 1000. Chronic toxicity was determined by the no observable effect concentration (*NOEC*), which can be 100, 50, or 10 mg/L for algae, invertebrates, and fish, respectively [31,32]. The risk quotient was then calculated by comparing the predicted no effect concentration (*PNEC*) with the measured environmental concentrations (*MEC*) of the herbicide of interest after the toxicity data was corrected by an assessment factor [30]:

(2) $$PNEC=\frac{EC_{50}}{AF}\;or\;PNEC=\frac{NOEC}{AF}\;$$

where *RQ* is the risk quotient calculated using the effective concentration (*EC*_50_) or no observable effect concentration (*NOEC*). The measured concentrations of the herbicides were represented by the *MEC*. The predicted no effect concentration (*PNEC*) was the highest concentration of a drug known to have no negative risks on organisms in the environment. The risks of herbicides in water sources were categorised as (i) low (*RQ* ≤ 0.1); (ii) medium (0.1 < *RQ* < 1); or (iii) high (*RQ* ≥ 1) [31,33].

**Table 4:** Acute and chronic toxicity data for detected herbicides in algae, invertebrate and fish.

Compound	Taxon	Specie	Acute Toxicity (*EC*_50_)	Chronic Toxicity (*NOEC*)	References
Atrazine	Algae	*P. Subcapitata*	0.059 mg/L	0.1 mg/L	[34]
	Invertebrate	*Daphnia magma*	6.9 mg/L	0.1 mg/L	[34]
	Fish	*Oncorhynchus mykiss*	4.5 mg/L	2 mg/L	[34]
Metolachlor	Algae	*P. Subcapitata*	57.1 mg/L	3.0 mg/L	[34]
	Invertebrate	*Daphnia magma*	23.5 mg/L	3.0 mg/L	[34]
	Fish	*Oncorhynchus mykiss*	3.9 mg/L	1.0 mg/L	[34]
Simazine	Algae	*P. Subcapitata*	0.04 mg/L	0.6 mg/L	[34]
	Invertebrate	*Daphnia magma*	1.1 mg/L	0.6 mg/L	[34]
	Fish	*Oncorhynchus mykiss*	9.0 mg/L	0.7 mg/L	[34]
Terbuthylazine	Algae	*P. Subcapitata*	0.02 mg/L	-	[35]
	Invertebrate	*Daphnia magma*	39.4 mg/L	0.21 mg/L	[35]
	Fish	*Oncorhynchus mykiss*	3.6 mg/L	0.13 mg/L ^a^	[35]

^a^ mysid shrimp.

### 2.4. Health Risk Assessment

The human health risk assessment method aids in appraising the likelihood and severity of adverse risks that a specific herbicide may pose in humans. It consists of various steps, such as hazard identification, exposure assessment, dose–response analysis, and risk characterisation [36,37,38]. The goal of the hazard identification step is to look into the type, concentration, and distribution of pesticides in a specific area [37]. The dose–response assessment step aids in determining the specific relationship between the contaminant exposure dose and the likelihood of adverse reactions in the exposed population [38]. The exposure assessment step assesses the amount, rate, and time of a human’s exposure to a contaminant. Ingestion remains a major risk among the various routes of exposure. The average daily dose (*ADD*) of pesticides ingested by adults and children is calculated using Equation (3). Because of their behavioural and physiological differences, it is calculated separately [36,37,39]:

(3) $$ADD_{ing}=\frac{C\times IngR\times EF\times ED\times CF}{BW\times AT}$$

*IngR* denotes the rate of ingestion for adults and children. The concentration of herbicides is *C*, the exposure duration is *ED*, the conversion factor is *CF*, the body weight is *BW*, the exposure frequency is *EF*, and the average time is *AT* (Table 5). The non-carcinogenic risk is then calculated by dividing the average daily doses in Equation (4) by a corresponding reference dose (*RfD*) in Table 6. Equation (5) is then used to calculate the hazard index (*HI*) [36,39]:

(4) $$HQ=\frac{ADD}{RfD}$$

(5) HI=∑HQ 

where if the hazard quotient or index is less than one, it indicates a very low risk; between one and four, it indicates a possible risk; and when greater than four, it indicates a high risk [36,39]. The *ADD* values in Equation (3) are also used to compute the cancer risk (*CR*) of each examined contaminant. Using Equation (6), the cancer risk is then assessed by multiplying the *ADD* by a corresponding slope factor (*SF*) in Table 6:

(6) CR=ADD×SF

The permitted risk ranges are <10^−6^ (very low risk), 10^−6^–10^−5^ (low risk), 10^−5^–10^−4^ (medium risk), 10^−4^–10^−3^ (high risk), and >10^−3^ (very high risk) [36,39].

**Table 5:** Exposure assessment parameters for ingestion pathway.

Parameters	Description	Unit	Values		Reference
			Adult	Children	
*BW*	Body weight	kg	70	28	[37,40]
*EF*	Exposure frequency	d/year	350	350	[36,39]
*ED*	Exposure duration	years	30	6	[36,39]
*IngR*	ingestion rate	L/day	2	1.5	[37,41]
*AT*	Average time (cancer)	days	365 × 70	365 × 70	[36,39]
	Average time(non-cancer)	days	365 × *ED*	365 × *ED*	[36,39]
*CF*	Conversion factor	L/cm^3^	0.003	0.003	[37,40]
*C*	Concentration	mg/L	-	-	-

**Table 6:** Herbicide reference doses and cancer slope factors.

Contaminant	Reference Doses (*RfD*)	Cancer Slope Factor (*CSF*)	Reference
Atrazine	0.0035 mg/kg-day	0.23 mg/kg-day	[42]
Metolachlor	0.15 mg/kg-day	0.0092 mg/kg-day	[42]
Simazine	0.005 mg/kg-day	0.12 mg/kg-day	[42]
Terbuthylazine	-	-	-

## 3. Results and Discussion

### 3.1. Pesticides in Water Resources

Table 7 displays the rate of detection and concentrations of pesticides found in this study. Minimum, maximum, average, and standard deviation were the summary for descriptive statistics. When a contaminant was detected in only one sample, no standard deviation was calculated. The findings were discussed in terms of their presence in rivers, dams/reservoirs, and treated drinking water. This study targeted four (4) herbicides: atrazine, simazine, terbuthylazine, and metolachlor. The pesticides discovered were classified as triazines (atrazine, simazine, and terbuthylazine) and chloroacetanilides (metolachlor). Triazine herbicides, such as atrazine, simazine, and terbuthylazine, and chloroacetanilide herbicides, such as metolachlor, were targeted in this study because agriculture dominates the landscape of Mangaung Metropolitan Municipality. These compounds are the most commonly used around the world and are associated with serious health effects [4]. The availability of their standards in the laboratory was also one of the influencing factors.

#### 3.1.1. Pesticides in Rivers

As shown in Table 7, all herbicides had 100% detection frequency, with the exception of simazine, which was detected in 80% of the 5 river samples collected. The mean concentrations of atrazine, metolachlor, simazine, and terbuthylazine were 0.03 mg/L, 0.01 mg/L, 1.82 mg/L, and 0.06 mg/L, respectively. Simazine had the highest concentration among the herbicides, despite being detected in 80% of the collected samples, while metolachlor had the lowest. The detection of pesticides in river water in this study is comparable to other published works, which detected herbicides, such as simazine, in Cape Town, South Africa [26], terbuthylazine in Western Cape, South Africa [24], metolachlor in Hungary [43], and atrazine in Maryland, United States [11]. Most of the rivers in this study pass through the city, industrial areas, and townships. They are also located near the roadside and agricultural fields. Therefore, the presence of herbicides in river water was not unexpected in this study, especially triazine herbicides, which were the most prevalent herbicides in river samples. Triazine herbicides are considered effective and low-cost compounds that are primarily used in crop production [5]. Herbicides are also used to control weeds in industrial areas, along roadsides, and in public squares [6]. The agricultural sector is the backbone of the economy in the Mangaung Metropolitan Municipality. The area is known for producing a lot of maize, soybeans, wheat, sorghum, sunflower, potatoes, groundnuts, and wool. All of these activities necessitate the use of weed control herbicides before, during, and after farming. In addition, the Mangaung Metropolitan Municipality has well-developed roads, public parks, industrial areas, and golf clubs. With all these noticeable areas, significant amounts of herbicides are applied to control weeds in paving, parks, golf courts, roadsides, buildings, and industrial areas. Runoff from agricultural fields, roads, public squares, golf clubs, and industrial areas may increase the concentrations of herbicides in rivers around the municipality. Moreover, trace amounts of herbicides in this study may be introduced into streams by wastewater effluents mostly discharged in rivers.

#### 3.1.2. Pesticides in Dams/Reservoirs

The detection rates of atrazine, simazine, terbuthylazine, and metolachlor in dams/reservoirs were 100%, 80%, 100% and 100%, respectively, as shown in Table 7. Their corresponding average concentrations were 0.02 mg/L, 0.12 mg/L, 0.03 mg/L, and 0.01 mg/L. The concentrations of these herbicides were trending as simazine > terbuthylazine > atrazine = metolachlor. Simazine had the highest mean concentration of any herbicides detected. The present study highlighted the occurrence of herbicides in dams, showing that water pollution by organic compounds is a serious issue. Curchod et al.’s [24] study in Western Cape, South Africa, found concentrations of simazine and terbuthylazine which were lower than the current results. In Eastern Goiás of Brazil [44], atrazine and metolachlor concentrations were lower than the findings of this study, while in the semiarid region of Argentina [9], the detected concentrations of atrazine and metolachlor were higher than the findings of this study. Herbicides are predominantly utilised in agricultural activities, but substantial quantities are also utilised in forestry, industrial areas, public areas, parks, golf courts, and sports grounds for weed control and maintenance [5,6]. Pandey et al. [45] also stated that these herbicides can be used to control invasive plants in water. As a result, the presence of these herbicides in dams/reservoirs may be attributed to their use to control aquatic weeds, such as algae and submerged weeds. Some dams/reservoirs are distinguished by well-kept large open spaces with lawns and turf grasses near bodies of water for picnics. Herbicides may be required to suppress and control annual and perennial broadleaf and grassy weeds in these large open spaces with lawn and turf grasses. As a result, runoff from these lawns and turf grass areas may contaminate dams with simazine, terbuthylazine, atrazine, and metolachlor. According to Wang et al. [5], herbicides can be useful prior to and after cultivation to restrict broadleaf and grassy weeds in agricultural fields. Given that the majority of the dams/reservoirs are surrounded by and located near agricultural fields, runoff from those sites may possibly be a contributing factor. The use of these herbicides in dams may endanger the aquatic life and water consumers [5]. Moreover, rivers that discharge their water in dams may also introduce trace amounts of herbicides in dams. This is because most rivers receive effluents from wastewater treatment works, which receive water from various areas and are a source of many pollutants, including herbicides.

#### 3.1.3. Pesticides in Treated Drinking Water

As presented in Table 7, a 100% detection rate for atrazine, metolachlor, and terbuthylazine was recorded, whereas a 50% detection rate was observed for simazine. The mean concentrations of atrazine, metolachlor, simazine and terbuthylazine were 0.02 mg/L, 0.01 mg/L, 0.03 mg/L and 0.02 mg/L, respectively. Although not detected in all samples, the concentration of simazine was higher than that of all the detected herbicides in treated drinking water. From their measured concentration, these herbicides were trending as simazine > atrazine = tebuthylazine > metolachlor. The occurrence of herbicides such as atrazine and terbuthylazine in treated drinking water was also reported by Odendaal et al. [46] in their study aimed at determining contaminants of emerging concern in drinking water in South Africa. Machete and Shadung [47] also reported the occurrence of atrazine and terbuthylazine in treated drinking water in the Vals and Renoster catchment, South Africa. The use of herbicides to control weeds in dams/reservoirs used as a source of water in water treatment works may be the source of these herbicides. Rivers that discharge their water into these dams may contain traces of herbicides, as they mostly receive wastewater effluents. Atrazine detection in water sources may also be connected to their persistent nature. Almberg et al. [48] reported that herbicides such as atrazine are persistent in soil and their transport to water, making it the most commonly detected pesticide in water sources. Moreover, the presence of pesticides in treated drinking water revealed that the methods used in selected water treatment works are incapable of removing these compounds, which include abstraction, macro/micro sieving, coagulation, flocculation, sedimentation, filtration, and disinfection. Despite the widespread use of pesticides in South Africa, many of the country’s registered pesticides products have not been re-evaluated since their initial approval [49]. Among the detected pesticides in this study, South Africa only has water quality guidelines for the protection of human health and aquatic environments against atrazine, which is 0.01 mg/L [49,50]. Atrazine concentration was above the Republic of South African (RSA) acceptable limit. In order to protect public health from serious health effects, the World Health Organisation (WHO) has also implemented pesticides guideline levels in drinking water [51]. The mean concentration of herbicides in this study were above their corresponding World Health Organisation guidelines, except metolachlor, which was equal to its corresponding value. These findings show a negative situation and a serious health concern for the Mangaung community because chronic pesticide exposure through water ingestion can have serious health implications [4]. Table 8 compares herbicide detection to the World Health Organisation and South African guideline values.

### 3.2. Environmental Risk Assessment

The mean values of measured environmental concentrations were utilised to measure the ecological effects of herbicides on aquatic organisms. The risks of contaminants with concentrations below the limit of quantification (LOQ) were not assessed. In the absence of effective concentration (*EC*) values, lethal concentration (*LC*_50_) values were used. In cases where no observed effect concentration (*NOEC*) values were unavailable, the lowest observed effect concentration (*LOEC*) values were used [19,52]. As three representative organisms of the aquatic environment, the predicted non-effect concentrations for algae, invertebrates, and fish were used. In the event of data gaps, different species and endpoints were included. The results of risk assessment based on three representative organisms are shown in Table 9.

The study looked at both acute and chronic toxicities on aquatic organisms. In all water media studied, the risk of toxicity ranged from medium to high. In terms of acute toxicity, atrazine and simazine posed high risks to all aquatic organisms in all water media. Furthermore, high terbuthylazine risks were noticed in all aquatic organisms in rivers. However, in dams/reservoirs and treated drinking water, it posed a high risk to algae and fish. Metolachlor posed a high risk only to fish in all water sources. Remarkably, simazine was found to pose the greatest ecological risk in rivers, with risk quotient (*RQ*) values of 45,500, 1820, and 202 for algae, invertebrates, and fish, respectively.

In chronic toxicity, simazine showed a high risk to all aquatic organisms in rivers and dams. In treated drinking water, its high risks were noticed for algae and invertebrates. Algae and invertebrates were also sensitive to atrazine, with risk quotient (*RQ*) values greater than one in all water media. Terbuthylazine posed high risks to invertebrates and fish in rivers. In dams/reservoirs, it only posed a high risk to fish, while in treated drinking water, its high risk was observed for invertebrates. Metolachlor did not show high risk to all aquatic life in all water media. Moreover, in treated drinking water, none of the herbicides posed a high risk to fish. The greatest environmental risk was observed in river water for simazine with risk quotient (*RQ*) values of 303, 182, and 26 for algae, invertebrates, and fish, respectively.

The high risks of herbicides, such as simazine, atrazine, and terbuthylazine in water resources show an undesirable condition for the aquatic ecosystem in the Mangaung Metropolitan Municipality. Furthermore, unobserved pesticides and their mixtures may pose greater risks than those observed in this study [19]. These outcomes are expected to have an undesirable effect on aquatic life and human beings. There is evidence showing that exposure to simazine may cause weight changes as well as effects on the serum and thyroid gland [53]. Terbuthylazine may have an effect on carp growth rate, early ontogeny, and antioxidant enzyme [49]. Atrazine exposure may increase the risk of cancer, reproductive problems, and antibiotic resistance. In animals, metolachlor can cause salivation, lacrimation, and convulsions [54,55]. Crop farming, which accounts for 67.5% of all agricultural activities in the Mangaung Metro, may have exacerbated herbicide concentrations in water resources, which lead to their high ecological risks. Therefore, these pesticides should be prioritised and included in municipal environmental monitoring plans. Furthermore, the qualitative screening of unknown pesticides should also be considered in the near future. This will help to obtain a full understanding of the pesticide contaminants and associated risks in water resources within the Mangaung Metro.

### 3.3. Human Health Risk Assessment

Water sources, such as rivers, dams/reservoirs, and treated drinking water, play an important role in the lives of people within the Mangaung Metropolitan Municipality. In rural areas under the authority of Mangaung, these water sources serve as a source of water for various domestic purposes, including drinking. Therefore, the health risks of pesticides were focused on oral ingestion. This is because ingestion remains a major risk among the various routes of exposure [36,37,39]. The risks of pesticides with unknown cancer slope factors were not assessed. Table 10 and Table 11 present the results of non-carcinogenic and carcinogenic risks, respectively.

Pesticides in the water resources of the Mangaung Metropolitan Municipality were assessed for human health risks. In terms of non-carcinogenic risk, all pesticides posed low risks to both adults and children, with hazard quotient values less than one (Table 10). These values indicate that the Mangaung Metropolitan Municipality community is free of non-carcinogenic effects caused by the identified herbicides (atrazine, simazine, and metolachlor) exposure in water resources. In terms of carcinogenic risk, all pesticides in the river water showed very low risk to adults and children, with the exception of simazine, which showed medium risk to adults (Table 11). Furthermore, the presence of all pesticides posed a very low risk to adults and low risk to children in dams and treated drinking water. Identified herbicides in this study may have serious health consequences for the community, as the water from dams/reservoirs and rivers in the Mangaung Metropolitan Municipality is used to support farmers and local communities. Treated drinking water is also used for sanitary and household purposes. Furthermore, while the level of risk in this study was low to medium, it may be harmful to the community in the near future due to the continuous introduction of these contaminants. Unintended contact, the bioaccumulation of pesticide residues in fish and locally grown crops, and biomagnification in the food chain can cause considerable risks to the community of the Mangaung Metropolitan Municipality as a result of water polluted by these compounds [19]. Furthermore, herbicide degradation may yield one or more complex transformation products that may be more tenacious or noxious than the original compound [56]. The various health effects associated with herbicide exposure include, but are not limited to, oxidative stress, cytotoxicity, dopaminergic effects, sexual maturation delays, breast cancer, reproductive, and endocrine effects [3]. Human health risk assessment studies of pesticides in water sources are critical in this community for regulatory purposes [19] as well as the protection of public health and the country’s limited water resources.

## 4. Conclusions

The current project was motivated by the dearth of data on the level of herbicides in water sources around the Mangaung Metropolitan Municipality and their associated environmental and health risks. To the best of our knowledge, this was the first study on the Mangaung Metropolitan Municipality to address such issues. The study revealed that herbicides are present in rivers, dams/reservoirs and treated drinking water in this area. The presence of these herbicides shows a possibility to cause high ecological risks and medium carcinogenic risks during spring season, demonstrating that aquatic and human beings may be affected by these contaminants. The presence of pesticides in water sources within the Mangaung Metropolitan Municipality is concerning because South Africa is a water-stressed country, and exposure to pesticides may cause serious effects. Therefore, it is strongly advised that interventions aimed at determining the source of contamination be implemented to safeguard water resources and the health of water consumers and aquatic organisms. The findings of the study may serve as an awareness to the community and relevant stakeholders on the environmental and public health risks of pesticide exposure. It will also aid in the development of municipal pollution management and risk-reduction strategies for herbicides and other toxic compounds. Some limitations of this study include the fact that it only focused on a few pesticide families, which should be expanded in future studies to obtain an overall understanding of the risks associated with them. Furthermore, the predicted non-effect concentration values are based on currently available data and may change as more reliable data become available.

## Figures and Tables

**Table 1:** Location of the sampling points within Mangaung Metropolitan Municipality.

Sample ID	Description	Coordinates		
		Longitude	Latitude	Elevation
River samples				
SWRS03	Near intense agricultural activities	29°06′9.6″	26°19′7.2″	1379 m
SWBS04	Close to residential area and WWTW	29°07′2.4‴	26°17′1.5″	1390 m
SWKOR05	Passes through agricultural farms	29°12′8.3″	25°31′04″	1334 m
SWMOR06	Near farms, railroad, and national road	29°09′39.3″	26°34′20.3″	1327 m
SWKLM07	Passes through a township and near WWTW	29°14′34.3″	26°40′26.2″	1373 m
Dam/Reservoirs samples				
SWMS01	It also serves as a resort	29°01′4.9″	26°24′2.7″	1344 m
SWMD02	Serve as a resort, conference centre	29°02′8.4″	26°27′5.8″	1354 m
SWKD08	Fishing activities, and farms nearby	29°53′03″	25°57′21″	1226 m
SWSD09	Near settlement and industrial activities	29°12′10‴	26°47′38″	1460 m
SWRUSD10	Near farms and residents	29°16′20″	26°37′00″	1370 m
Treated drinking water sample				
TWWRU01	Water treatment works 1	29°16′31″	26°37′51″	-
TWWMSP02	Water treatment works 2	29°01′10.3″	26°24′9.2″	-

**Table 2:** Multiple reaction monitoring transition values for targeted compounds.

Analyte	Positive Ionisation Mode		Negative Ionisation Mode		
	Q1 (*m*/*z*)	Q3 (*m*/*z*)	Q1 (*m*/*z*)	Q3 (*m*/*z*)	Retention Time (Minutes)
Atrazine 1	216.049	174.2	216.049	174.2	21.80
Atrazine 2	216.049	68.1	216.049	68.1	21.80
Metolachlor 1	284.347	252	284.347	252	25.30
Metolachlor 2	284.347	176.2	284.347	176.2	25.30
Simazine 1	202.039	132.1	202.039	132.1	19.80
Simazine 2	202.039	104.1	202.039	104.1	19.80
Terbuthylazine 1	230.087	174.3	230.087	174.3	24.00
Terbuthylazine 2	230.087	68	230.087	68	24.00

**Table 3:** Linearity, and limit of quantification of the targeted analytes.

Analyte	Linearity (r-Value)	LOQ
Simazine	0.99	0.0100
Atrazine	0.99	0.0001
Terbuthylazine	0.99	0.0001
Metolachlor	0.99	0.0001

**Table 7:** Herbicides concentrations in rivers, dams/reservoirs and treated drinking water.

Concentration (mg/L)			
Compounds	DF (%)	Min-Max	Mean ± SD
Rivers (*n* = 5)			
Atrazine	100	0.002–0.06	0.03 ± 0.03
Metolachlor	100	0.003–0.03	0.01 ± 0.01
Simazine	80	<LOQ-5.67	1.82 ± 2.66
Terbuthylazine	100	0.01–0.21	0.06 ± 0.09
Dams/Reservoirs (*n* = 5)			
Atrazine	100	0.01–0.03	0.02 ± 0.01
Metolachlor	100	0.002–0.03	0.01 ± 0.01
Simazine	80	<LOQ-0.20	0.12 ± 0.08
Terbuthylazine	100	0.01–0.06	0.03 ± 0.02
Treated drinking water (*n* = 2)			
Atrazine	100	0.015–0.02	0.02 ± 0.003
Metolachlor	100	0.009–0.01	0.01 ± 0.001
Simazine	50	<LOQ-0.03	0.03
Terbuthylazine	100	0.019–0.02	0.02 ± 0.001

Notation: DF = detection frequency, *n* = number of samples, min = minimum concentration, max = maximum concentration, mg/L = milligram per litre, SD = standard deviation.

**Table 8:** Concentration of pesticides in treated drinking water with guidelines values [49,51].

Pesticides	Guideline Value		Concentration (mg/L)		Reference
	RSA (mg/L)	WHO (mg/L)	Maximum	Mean	
Atrazine	0.01	0.002	0.019	0.02	[49,51]
Metolachlor	-	0.01	0.01	0.01	[51]
Simazine	-	0.002	0.03	0.03	[51]
Terbuthylazine	-	0.007	0.02	0.02	[51]

**Table 9:** Ecological risks of pesticides in water resources.

Contaminant	Taxonomic Class	Acute Toxicity		Chronic Toxicity	
		HQ Mean	Risk	HQ Mean	Risk
River					
Atrazine	Algae ^a^	508	High	30	High
	Invertebrate ^b^	4.35	High	15	High
	Fish ^c^	6.67	High	0.15	Medium
Metolachlor	Algae ^a^	0.17	Medium	0.33	Medium
	Invertebrate ^b^	0.5	Medium	0.17	Medium
	Fish ^c^	2.5	High	0.1	Medium
Simazine	Algae ^a^	45,500	High	303	High
	Invertebrate ^b^	1820	High	182	High
	Fish ^c^	202	High	26	High
Terbuthylazine	Algae ^a^	3000	High	-	-
	Invertebrate ^b^	1.54	High	14.28	High
	Fish ^c^	16.67	High	46.15	High
Dams/Reservoirs					
Atrazine	Algae ^a^	339	High	20	High
	Invertebrate ^b^	2.9	High	10	High
	Fish ^c^	4.44	High	0.1	Medium
Metolachlor	Algae ^a^	0.17	Medium	0.33	Medium
	Invertebrate ^b^	0.5	Medium	0.17	Medium
	Fish ^c^	2.5	High	0.1	Medium
Simazine	Algae ^a^	2500	High	16.67	High
	Invertebrate ^b^	100	High	10	High
	Fish ^c^	11	High	1.43	High
Terbuthylazine	Algae ^a^	1500	High	-	-
	Invertebrate ^b^	0.77	Medium	0.71	Medium
	Fish ^c^	8.33	High	2.31	High
Treated drinking water					
Atrazine	Algae ^a^	339	High	20	High
	Invertebrate ^b^	2.89	High	10	High
	Fish ^c^	4.44	High	0.1	Medium
Metolachlor	Algae ^a^	0.17	Medium	0.33	Medium
	Invertebrate ^b^	0.5	Medium	0.17	Medium
	Fish ^c^	2.5	High	0.1	Medium
Simazine	Algae ^a^	750	High	5	High
	Invertebrate ^b^	30	High	3	High
	Fish ^c^	3.33	High	0.43	Medium
Terbuthylazine	Algae ^a^	1000	High	-	-
	Invertebrate ^b^	0.77	Medium	4.76	High
	Fish ^c,d^	5.55	High	0.15	Medium

Notation: HQ: hazard quotient. Acute and chronic toxicity data of selected species were extracted from data available in the literature. ^a^ *P. subcapitata;* ^b^ *Daphnia magna*; ^c^ *Oncorhynchus mykiss*; ^d^ *Mysid shrimp* [34,35].

**Table 10:** Non-carcinogenic risks of pesticides in water resources.

Compound	Rivers		Dams/Reservoir		Treated Drinking Water	
	ADD	HQ	ADD	HQ	ADD	HQ
Adults						
Atrazine	8.2 × 10^−7^	2.3 × 10^−7^	5.5 × 10^−7^	1.6 × 10^−4^	5.5 × 10^−7^	1.6 × 10^−4^
Metolachlor	2.7 × 10^−7^	1.8 × 10^−6^	2.7 × 10^−7^	1.8 × 10^−6^	2.7 × 10^−7^	1.8 × 10^−6^
Simazine	5 × 10^−5^	1.6 × 10^−2^	2.7 × 10^−6^	5.4 × 10^−4^	8.2 × 10^−7^	1.6 × 10^−4^
Terbuthylazine	1.6 × 10^−6^	-	8.2 × 10^−7^	-	5.5 × 10^−7^	-
Total	5.3 × 10^−5^	1.6 × 10^−2^	4.3 × 10^−6^	7 × 10^−4^	2.2 × 10^−6^	3.2 × 10^−4^
Children						
Atrazine	1.5 × 10^−6^	4.3 × 10^−4^	4.1 × 10^−7^	1.2 × 10^−4^	4.1 × 10^−7^	1.2 × 10^−4^
Metolachlor	5 × 10^−7^	3.3 × 10^−6^	2 × 10^−7^	1.3 × 10^−6^	2 × 10^−7^	1.3 × 10^−6^
Simazine	9.3 × 10^−5^	1.9 × 10^−2^	2.1 × 10^−6^	4.2 × 10^−4^	6.1 × 10^−7^	1.2 × 10^−4^
Terbuthylazine	3.1 × 10^−6^	-	6.1 × 10^−7^	-	4.1 × 10^−7^	-
Total	9.8 × 10^−5^	1.9 × 10^−2^	3.3 × 10^−6^	5.4 × 10^−4^	1.6 × 10^−7^	2.4 × 10^−4^

Notation: ADD: average daily dose; HQ: hazard quotient.

**Table 11:** Carcinogenic risks of pesticides in water resources.

Compound	Rivers		Dams/Reservoir		Treated Drinking Water	
	ADD	CR	ADD	CR	ADD	CR
Adults						
Atrazine	3.5 × 10^−7^	8 × 10^−8^	2.3 × 10^−7^	5.3 × 10^−8^	2.3 × 10^−7^	5.3 × 10^−8^
Metolachlor	1.2 × 10^−7^	1.1 × 10^−9^	1.2 × 10^−5^	1.1 × 10^−7^	1.2 × 10^−7^	1.1 × 10^−9^
Simazine	2.1 × 10^−5^	2.5 × 10^−6^	1.2 × 10^−6^	1.4 × 10^−7^	3.5 × 10^−8^	4.2 × 10^−9^
Terbuthylazine	7 × 10^−7^	-	3.5 × 10^−7^	-	2.3 × 10^−7^	-
Total	2.2 × 10^−5^	2.5 × 10^−6^	1.4 × 10^−5^	3 × 10^−7^	6.5 × 10^−7^	5.8 × 10^−8^
Children						
Atrazine	1.3 × 10^−7^	2.9 × 10^−8^	8.8 × 10^−8^	2 × 10^−8^	8.8 × 10^−8^	2 × 10^−8^
Metolachlor	4.3 × 10^−8^	3.9 × 10^−10^	4.3 × 10^−8^	3.9 × 10^−10^	4.3 × 10^−8^	3.9 × 10^−10^
Simazine	8 × 10^−6^	9.6 × 10^−7^	4.3 × 10^−7^	5.2 × 10^−8^	1.3 × 10^−8^	1.5 × 10^−9^
Terbuthylazine	2.6 × 10^−7^	-	1.2 × 10^−7^	-	4.3 × 10^−7^	-
Total	8.4 × 10^−6^	9.9 × 10^−7^	6.8 × 10^−7^	7.2 × 10^−8^	5.7 × 10^−7^	2.2 × 10^−8^

Notation: ADD: average daily dose; CR: cancer risk.

## Data Availability

Available on request from the corresponding author.
